# Mps1 kinase-dependent Sgo2 centromere localisation mediates cohesin protection in mouse oocyte meiosis I

**DOI:** 10.1038/s41467-017-00774-3

**Published:** 2017-09-25

**Authors:** Warif El Yakoubi, Eulalie Buffin, Damien Cladière, Yulia Gryaznova, Inés Berenguer, Sandra A. Touati, Rocío Gómez, José A. Suja, Jan M. van Deursen, Katja Wassmann

**Affiliations:** 1 Sorbonne Universités, UPMC Univ. Paris 06, Institut de Biologie Paris Seine (IBPS), UMR7622, Paris, 75005 France; 2 CNRS, IBPS, UMR7622 Developmental Biology Lab, Paris, 75005 France; 3 Unidad de Biología Celular, Departamento de Biología, Facultad de Ciencias, Universidad Autónoma de Madrid, 28049 Madrid, Spain; 4 Department of Pediatric and Adolescent Medicine and Department of Biochemistry and Molecular Biology, Mayo Clinic College of Medicine, Rochester, MN 55905 USA; 5 Present Address: Chromosome Segregation Laboratory, Lincoln’s Inn Fields Laboratory, The Francis Crick Institute, London, WC2A 3LY UK

## Abstract

A key feature of meiosis is the step-wise removal of cohesin, the protein complex holding sister chromatids together, first from arms in meiosis I and then from the centromere region in meiosis II. Centromeric cohesin is protected by Sgo2 from Separase-mediated cleavage, in order to maintain sister chromatids together until their separation in meiosis II. Failures in step-wise cohesin removal result in aneuploid gametes, preventing the generation of healthy embryos. Here, we report that kinase activities of Bub1 and Mps1 are required for Sgo2 localisation to the centromere region. Mps1 inhibitor-treated oocytes are defective in centromeric cohesin protection, whereas oocytes devoid of Bub1 kinase activity, which cannot phosphorylate H2A at T121, are not perturbed in cohesin protection as long as Mps1 is functional. Mps1 and Bub1 kinase activities localise Sgo2 in meiosis I preferentially to the centromere and pericentromere respectively, indicating that Sgo2 at the centromere is required for protection.

## Abstract (web summary)

In meiosis I centromeric cohesin is protected by Sgo2 from Separase-mediated cleavage ensuring that sister chromatids are kept together until their separation in meiosis II. Here the authors demonstrate that Bub1 and Mps1 kinase activities are required for Sgo2 localisation to the centromere region.

## Introduction

Haploid gametes must harbour the correct number of chromosomes for successful embryo development^1^. They are generated through two successive meiotic divisions, meiosis I and II^2, 3^. In meiosis I homologous chromosomes are segregated, and in meiosis II, sister chromatids. Proper execution of the meiotic divisions depends on the step-wise removal of cohesin, which is holding paired sister chromatids together. Cohesin is a multi-protein complex localised to centromeres and chromatid arms. In meiosis, centromeric cohesin maintains sister chromatids together, and arm cohesin stabilizes chiasmata (sites of recombination) holding homologous chromosomes together. At metaphase-to-anaphase transition, cleavage of cohesin’s kleisin subunit Rec8 by Separase removes the cohesive forces exerted by cohesin. For the segregation of homologous chromosomes in meiosis I, cohesin is removed from chromosome arms, and maintained in the centromeric region where it is protected from Separase-dependent cleavage. In meiosis II, centromeric cohesin is removed and it is only then that sister chromatids can separate to generate haploid gametes^4–6^. The protection of centromeric cohesin in meiosis I is therefore essential to prevent precocious sister chromatid separation and generation of aneuploid gametes.

In male and female meiosis Shugoshin2 (Sgo2) localisation to the centromere is essential for protection of cohesin^7–9^. Without Sgo2, bivalent chromosomes are still correctly oriented and chromosomes segregate in meiosis I, but then sister chromatids fall apart in anaphase I because they are no longer maintained together by centromeric cohesin. As a consequence, no tension bearing attachments can be established in metaphase of meiosis II, and sister chromatids segregate at random in anaphase II. Sgo2 knock-out mice are therefore unable to generate gametes of correct ploidy, and are sterile^7^.

Sgo2 mediates protection of centromeric cohesin in meiosis I through recruitment of the phosphatase PP2A-B56^4^. It is thought that analogous to yeast, PP2A-B56 maintains the meiotic cohesin subunit Rec8 dephosphorylated and thereby non-cleavable for Separase in mammals^9–12^. In oocyte meiosis II, Sgo2-PP2A is still recruited to the centromere, but before anaphase onset, tension applied by the bipolar spindle and co-localisation of I2PP2A/Set with PP2A-B56 antagonise centromeric cohesin protection to promote Separase-dependent removal of cohesin^8, 13–15^.

It is poorly understood how Sgo2 protein is recruited to the centromere in meiosis. Sgo1, which is related to Sgo2, protects cohesin from removal by the so-named prophase pathway in mitosis^16, 17^. Recruitment of Sgo1 takes place through Bub1 kinase-dependent phosphorylation of Histone H2A on Threonine 120 (H2A-pT120)^17–21^. Whether this is also the mechanism of Sgo2 recruitment in meiosis is still elusive.

Spindle assembly checkpoint (SAC) components have been shown to play important roles during mitotic and meiotic cell division in addition to their well-characterised roles for SAC control^22–26^. Mps1 and Bub1 kinases are essential for meiotic SAC control, but whether they are required for cohesin protection in meiosis was unknown. Bub1 knock-out oocytes separate some but not all sister chromatids before metaphase II, indicating that Bub1 participates, but is not the only factor for Sgo2 localisation and cohesin protection^27^. Mice harbouring only a kinase-dead allele of Bub1 are not sterile, demonstrating that Bub1 phosphorylation of Histone H2A is not absolutely required to generate healthy gametes^28^. The SAC kinase Mps1 was shown in mitosis to be required for Bub1 kinetochore localisation and efficient H2A phosphorylation to recruit Sgo1^29, 30^, but chemical inhibition of Mps1 had only a minor effect on mitotic Sgo2 localisation, indicating that Sgo2 is localised differently from Sgo1 in mitosis^29^. Bub1’s autophosphorylation and kinase activity are thought to be essential for focused Sgo1 but once again not for Sgo2 recruitment in mitosis^21^. Mps1’s and Bub1’s potential roles for Sgo2 localisation and centromeric cohesin protection were therefore unknown. Their involvement in Sgo2 recruitment was important to be clarified in meiosis, where Sgo2 is essential for centromeric cohesin protection and the generation of euploid gametes.

By combining mouse genetics, knock-down approaches, and chemical inhibitors with in vitro oocyte culture we show here that kinase activities of both, Mps1 and Bub1, are required for efficient Sgo2 localisation to the centromere region. Using optimised confocal imaging we found that there are at least two distinguishable pools of Sgo2 in oocyte meiosis I: one at the centromere and the other one between sisters within the pericentromere. Importantly, Sgo2 at the centromere is required for protection of centromeric cohesin. Mps1 kinase activity but not kinetochore enrichment is required for correctly localising Sgo2 to the centromere, and in its absence, precocious sister chromatid separation in meiosis I occurs. Additionally, Mps1-dependent Bub1 localisation creates the H2A phosphorylation mark for recruitment of Sgo2 within the pericentromere, but this pool of Sgo2 is not essential for protection.

## Results

### Inhibition of Mps1 kinase activity causes sister chromatid separation

**Fig. 1:**
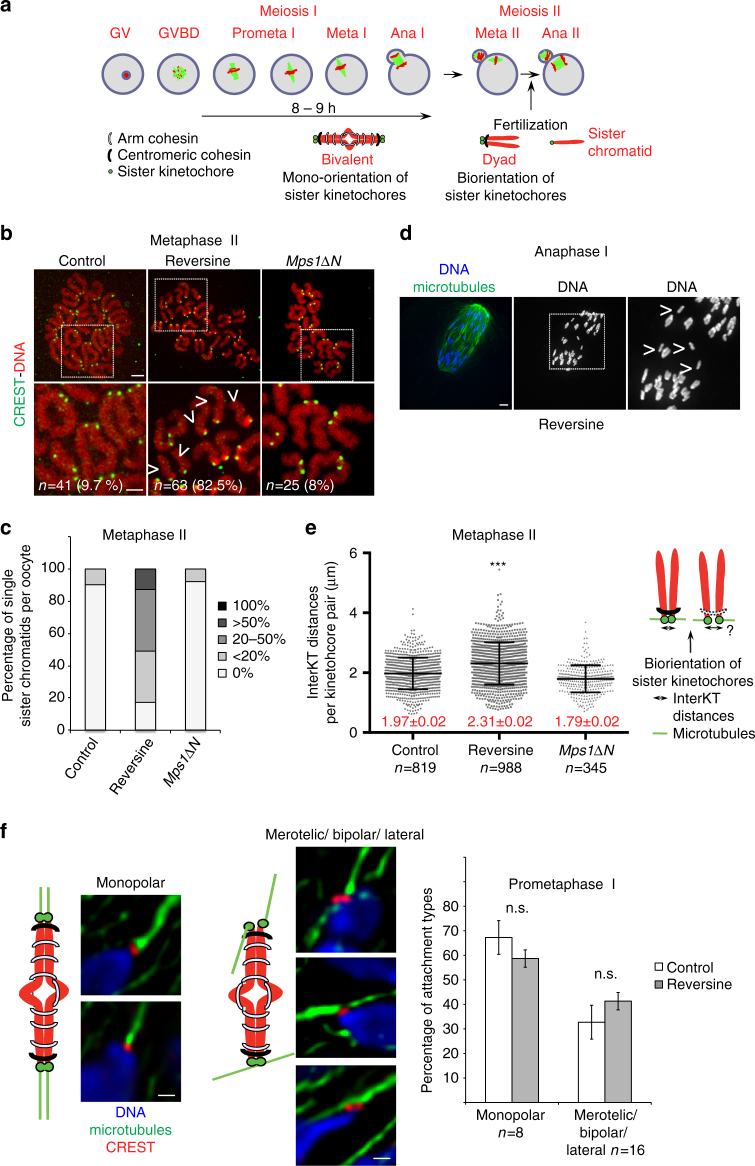
Loss of Mps1 kinase leads to increased interKT distances and presence of single sister chromatids in metaphase II. **a** Scheme of meiotic maturation in mouse oocytes. Spindles are in *green*, chromosomes in *red*. Corresponding chromosome figures are shown below the oocytes. GV, germinal vesicle; GVBD, germinal vesicle breakdown; Prometa, prometaphase; Meta, metaphase; Ana, anaphase. **b** Control oocytes, oocytes treated with Reversine (from GVBD onwards) and *Mps1∆N* oocytes were fixed for chromosome spreads after PB extrusion in meiosis II. *n* number of oocytes analysed. Percentage of oocytes with single sisters is indicated. Chromosomes were stained with Propidium iodide (*red*), kinetochores with CREST (*green*). Images below are magnification of the region indicated by the *white square*. *Arrowheads* indicate single sisters. **c** Percentage of metaphase II oocytes containing 0%, <20%, between 20 and 50%, more than 50 or 100% of single sister chromatids, observed in oocytes from **b** in at least three independent experiments. **d** Oocytes treated with Reversine (from GVBD onward) were fixed around the time of PB extrusion. Whole mount oocytes were stained with anti-tubulin antibody and Hoechst, to visualise spindles and DNA (*green* and *blue*, respectively). Shown is an example of an oocyte in anaphase I. The image on the right is a magnification of the region indicated by the *white square*. *Arrowheads* indicate single sisters. **e** Schemes of interKT distance between two bioriented sister kinetochores with normal or less cohesion in metaphase II are shown on the right. Quantification of interKT distances in metaphase II control oocytes, Reversine-treated oocytes and *Mps1∆N* oocytes is shown on the left. Only kinetochores of intact dyads (not already separated) were used for measurements. *n* number of kinetochore pairs analysed. Mean and *error bars* ± s.d. are indicated. Mean values are indicated in *red* for each condition. (***P < 0.0001) **f** On the *left*: schemes of monopolar and merotelic/lateral/bipolar attached sister kinetochores with corresponding images observed in whole mount oocytes fixed at GVBD + 4h after cold treatment. Microtubules are stained with anti-tubulin (*green*), sister kinetochores with CREST (*red*), and DNA with Hoechst (*blue*). On the *right*: percentage of monopolar and merotelic/lateral/bipolar sister kinetochore attachments observed. *n* number of oocytes analysed. Values indicate mean, and error bars are ± s.e.m. (*n.s*. not significant). *Scale bars*: 5 μm (**b**, **d**) and 0.5 µm (**f**)

To address whether Mps1 kinase is required for additional aspects in oocyte meiosis apart from its role in the SAC, we employed a chemical approach to target the kinase activity of Mps1 as oocytes enter meiosis I. Mouse oocytes obtained from adult mice are arrested at the Germinal vesicle (GV) stage, which corresponds to prophase of meiosis I. In culture, GV oocytes can be induced to enter meiosis I. They undergo the first meiotic division in a very synchronized manner and progress into metaphase of meiosis II where they await fertilisation A key feature of meiosis is the mono-orientation of sister kinetochores in meiosis I, and biorientation of sister kinetochores in meiosis II. Chiasmata and cohesion on chromosome arms maintain chromosomes together in meiosis I, and cohesion in the centromere region keeps sister chromatids together as oocytes progress into meiosis II until their separation in anaphase II (Fig. 1a).

GV oocytes were harvested and induced to enter meiosis I (visualised through germinal vesicle breakdown, GVBD) before addition of the well-characterised Mps1 kinase inhibitor Reversine^31^. Progression through the first meiotic division with polar body (PB) extrusion in the presence of Reversine was analysed. As we had shown previously, anaphase I onset takes place earlier and SAC control was abrogated in the presence of Reversine, similar to oocytes harbouring a conditional knock-out allele of any of the SAC components^27, 32, 33^, demonstrating that Reversine efficiently inhibits Mps1 kinase activity in oocytes^33^. Surprisingly though, when we performed chromosome spreads immediately after the first meiotic division, single sister chromatids were observed in 82% of oocytes in this strain background (Fig. 1b). In total, 31% of oocytes present < 20% of single sister chromatids, 38% of oocytes present 20–50% of single sister chromatids, and 13% of oocytes present more than 50% of single sister chromatids (Fig. 1c). Single sisters were sometimes also visible in whole mount fixed oocytes undergoing anaphase I (Fig. 1d). This indicates that some sister chromatids separated precociously in the absence of Mps1 kinase activity, suggesting that monopolar attachment of sister kinetochores, and/or centromeric cohesin was lost in meiosis I oocytes treated with Reversine.

We asked whether oocytes harbouring only a mutant form of Mps1 (‘*Mps1ΔN*’) which cannot localise correctly to kinetochores, and whose checkpoint is completely impaired^32^, but which still harbour Mps1 kinase activity, also separated sister chromatids before meiosis II. *Mps1ΔN* oocytes undergo metaphase-to-anaphase transition at the same time as Reversine-treated oocytes, and also with chromosomes that are not yet correctly aligned at the metaphase plate^32, 33^. Importantly, no increase in the presence of single sister chromatids was observed in *Mps1ΔN* metaphase II oocytes (Fig. 1b, c).

### Increased inter-kinetochore distances without Mps1 kinase activity

To determine whether centromeric cohesion which has to be maintained throughout the first division, was reduced as oocytes progress into meiosis II we compared inter-kinetochore (inter-KT) distances in metaphase II oocytes with or without Reversine treatment. Increased interKT distances of sister chromatids were for example observed in oocytes of aged mice, indicating loss of cohesion with age^34, 35^. Indeed, interKT distances of intact dyads (sister chromatid pairs that were not already separated upon Reversine treatment) were significantly increased compared to control oocytes (Fig. 1e). On the other hand *Mps1ΔN* oocytes, which did not precociously segregate sister chromatids, did not show increased interKT distances in metaphase II (Fig. 1e). This suggests that loss of cohesion in the centromere region is responsible for the observed precocious separation of sister chromatids in Reversine-treated oocytes.

### Early anaphase I onset does not lead to precocious sister separation

Oocytes without Sgo2 segregate bivalents correctly in meiosis I, because attachments of kinetochore pairs are still monopolar^7^. The presence of single sisters in anaphase I in Reversine-treated oocytes may therefore indicate defects in monopolar attachments leading to loss of centromeric cohesion. To determine whether defects in mono-orientation of sister kinetochores in meiosis I were responsible for the observed precocious separation of sister chromatids, we asked how paired sister kinetochores of bivalents were attached in Reversine-treated oocytes before PB extrusion, compared to controls at the same time. It has been shown previously that kinetochores in oocytes undergo multiple attachment and detachment cycles in prometaphase I^36^. Indeed, we observed a high proportion of merotelic and lateral attachments in prometaphase of meiosis I, independent of whether oocytes were treated with Reversine, or not (Fig. 1f). Therefore, Reversine treatment does not lead to a further increase of wrongly attached kinetochores. But early anaphase onset in oocytes deficient for SAC control, at a time when a high proportion of merotelic attachments are present, may indeed lead to the segregation of sister chromatids in meiosis I. In this case we would expect that *Mps1ΔN* oocytes, which undergo anaphase I at the same time as Reversine-treated oocytes, to precociously segregate sister chromatids as well, which was not the case (Fig. 1b, c). Oocytes devoid of BubR1, another essential SAC component causing accelerated anaphase I onset, showed no single sister chromatids in metaphase II either (Supplementary Fig. 1a). Therefore we conclude that early anaphase I onset at a time when microtubule fibres are still undergoing multiple attachment and detachment cycles with lateral, bipolar, and merotelic attachments was not the reason for the presence of single sisters in Mps1-inhibitor-treated oocytes. Nevertheless, we do not exclude that bipolar attachments at the time of anaphase I onset enhance the phenotype observed in Reversine-treated oocytes. Importantly, the presence of bipolar attachments at the time of anaphase I onset in combination with a loss of centromeric cohesion may explain the presence of single sister chromatids on anaphase I spindles we observed (Fig. 1d), and which does not occur in Sgo2 knock-out oocytes that are delayed in anaphase I onset^9^.

To exclude that the phenotype observed in Reversine was due to an off-target effect of the drug, we analysed oocytes subjected to Mps1 morpholino-mediated knock-down. Knock-down efficiencies were controlled in individual oocytes by immunostaining for Mps1 at kinetochores (Supplementary Fig. 1b). Importantly, even though Mps1 protein levels were only reduced at kinetochores, knock-down oocytes showed precocious separation of sister chromatids (Supplementary Fig. 1c, d) and increased interKT distances in meiosis II (Supplementary Fig. 1e), thus confirming that functional Mps1 kinase is required for maintaining sister chromatids together.

### Sgo2 localisation in meiosis I partially depends on Mps1 kinase

**Fig. 2:**
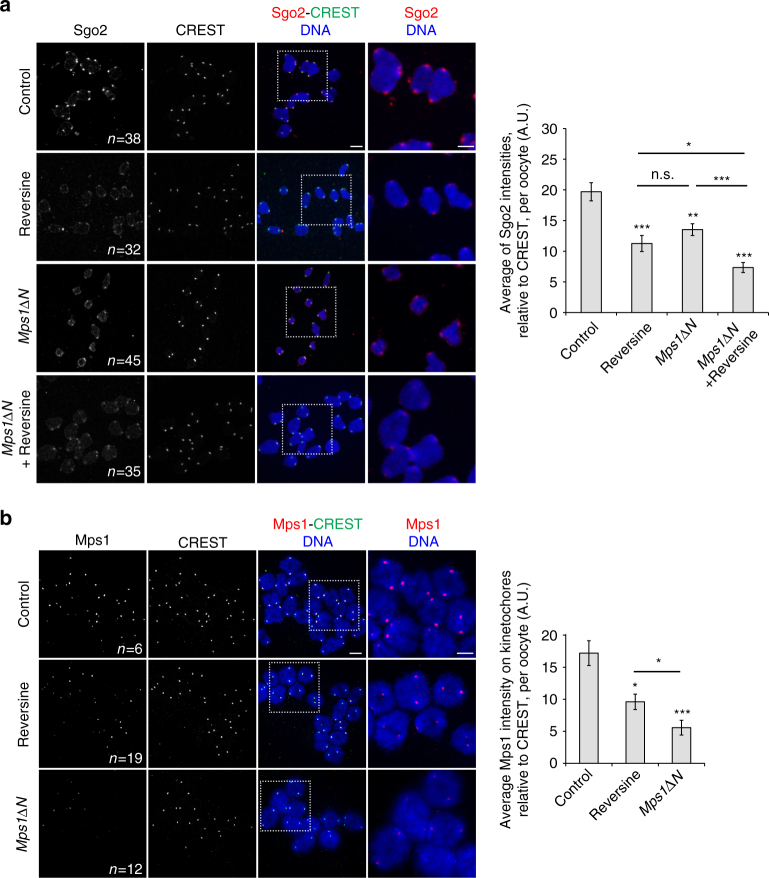
Mps1 kinase activity contributes to Sgo2 centromere recruitment. **a** Chromosomes spread performed at GVBD + 3h30 of control and *Mps1ΔN* oocytes treated or not with Reversine (from GVBD onward), and stained with antibodies against Sgo2 (*red*) and CREST serum (*green*). Chromosomes were stained with Hoechst (*blue*). On the right the corresponding quantification of the Sgo2 signal relative to CREST staining is shown. **b** Chromosomes spread performed at GVBD + 3h30 of control, Reversine-treated, and *Mps1∆N* oocytes and stained with antibodies against Mps1 (*red*) and CREST serum (*green*). Chromosomes were stained with Hoechst (*blue*). On the right the corresponding quantification of the Mps1 signal relative to CREST staining is shown. In **a**, **b**, the images on the right are magnification of the region indicated by the *white square*. *n* number of oocytes analysed. In each histogram, values indicate mean, *error bars* ± s.e.m., from six (**a**) or three (**b**) independent experiments, using Student’s *t*-test. (*n.s*. not significant; *P < 0.05, **P < 0.001, ***P < 0.0001). A.U., arbitrary units. *Scale bars*: 5 μm

According to Mps1 immunostaining on chromosome spreads, Mps1 was localised to kinetochores in prometaphase and metaphase I, was strongly reduced in anaphase I, and came back to kinetochores as oocytes progressed into meiosis II (Supplementary Fig. 2a). We hypothesised that Mps1 is required for some aspects of centromeric cohesin protection in meiosis I, leading to the observed phenotypes in Reversine-treated oocytes. Precocious removal of centromeric cohesin in Sgo2 knock-out oocytes and spermatocytes leads to separation of sister chromatids in meiosis I instead of meiosis II^7, 9^. Therefore Mps1 may be required for Sgo2-dependent protection of centromeric cohesin. We asked whether Sgo2 was correctly localised to the centromeric region in metaphase I without functional Mps1. Sgo2 localisation was significantly reduced in oocytes treated with Reversine, but also in *Mps1ΔN* oocytes. Treating *Mps1ΔN* oocytes with Reversine additionally increased loss of Sgo2 from the centromere region, probably due to additional loss of Mps1 kinase activity from kinetochores (Fig. 2a, Supplementary Fig. 2b shows the quantifications per kinetochore). Importantly, amounts of kinetochore-localised Mps1 are not directly linked to the amount of Sgo2 at the centromere, indicating the importance of active Mps1 for Sgo2 recruitment (Fig. 2b). Morpholino-mediated knock-down of Mps1 also lead to a reduction of Sgo2 (Supplementary Fig. 3a). However, Sgo2 reduction and the percentage of precocious sister separation (Fig. 1d) are significantly lower than in Reversine-treated oocytes, because of weak efficiency of the knock-down (Supplementary Fig. 1b). *BubR1*−/− oocytes which did not harbour single sister chromatids (Supplementary Fig. 1a) even though anaphase onset is strongly accelerated, showed no reduction of Sgo2 levels in the centromere region (Supplementary Fig. 3b). To ascertain that the reduction of Sgo2 in Reversine-treated oocytes is not an artefact of accelerated progression through meiosis I, we prolonged prometaphase with the proteasome inhibitor MG132 and compared Sgo2 levels in metaphase I. Reversine-treated oocytes again showed a reduction in Sgo2 levels when compared to controls, as shown above for oocytes in prometaphase I (Supplementary Fig. 3c). Thus, Mps1 kinase activity is required for Sgo2 localisation and thereby protection of centromeric cohesin, in a manner independent of its role in the activation of the SAC, and independent of the time of anaphase I onset.

Mps1 kinase activity may be required for recruitment of Sgo2, or alternatively, for maintenance of Sgo2 in the centromere region. To address this point oocytes were treated with Reversine in late prometaphase (GVBD + 4h) and analysed for Sgo2 localisation to the centromeric region at GVBD + 5.5h. Sgo2 amounts were indistinguishable from control oocytes **(**Supplementary Fig. 3d), therefore we conclude that Mps1 kinase is required for initial recruitment of Sgo2 to the centromere region, and not maintenance.

### Bub1 kinase activity is not essential for cohesin protection

**Fig. 3:**
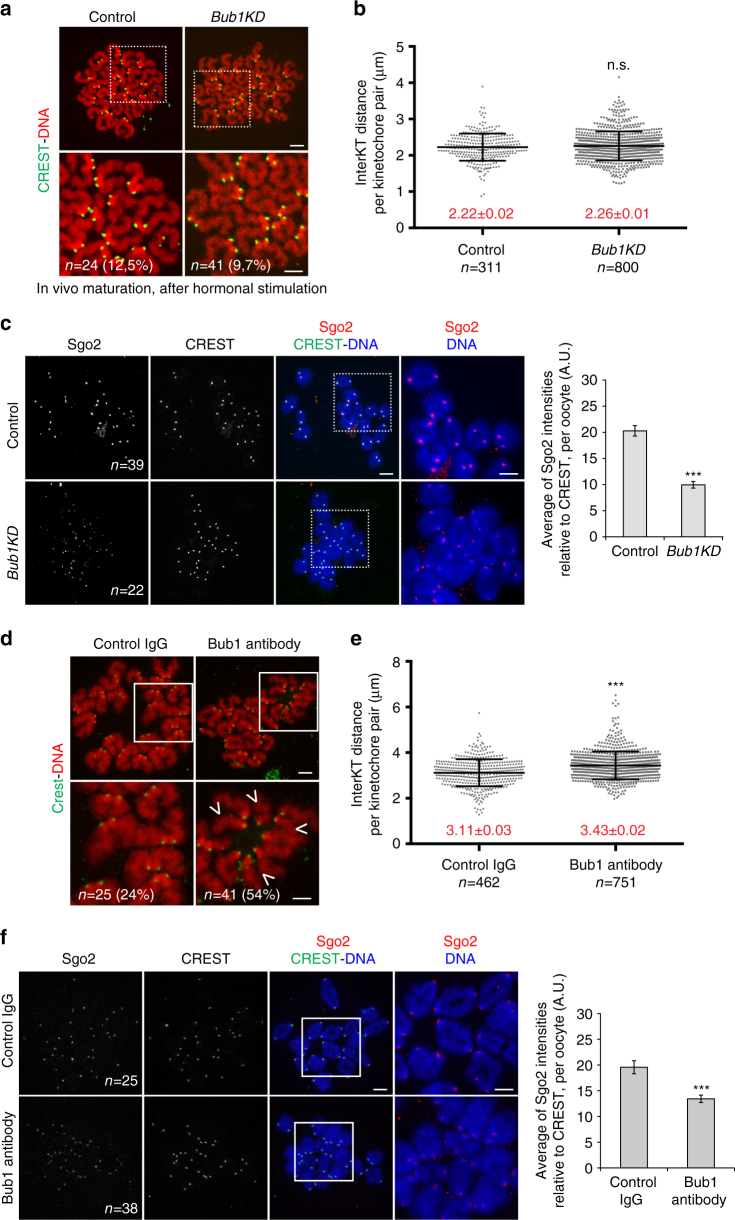
Bub1 kinase activity contributes to Sgo2 recruitment but is not essential for cohesin protection in meiosis I. **a** Chromosome spreads of oocytes from control and *Bub1KD* mice performed in metaphase II. Oocytes were matured in vivo after hormonal stimulation and harvested in metaphase II. The number of oocytes analysed is indicated, as well as the percentage of oocytes with single sisters. Chromosomes were stained with Propidium iodide (*red*), kinetochores with CREST (*green*). Images below are magnifications of the region indicated by the *white square*. **b** Quantification of interKT distances of oocytes in **a**, as described in Fig. 1. *n* number of kinetochore pairs analysed. No significant difference was observed. **c** Chromosome spreads performed at GVBD + 3h30 of control and *Bub1KD* oocytes and stained with anti-Sgo2 antibody (*red*), and CREST anti-serum (*green*). Chromosomes were stained with Hoechst (*blue*). The number of oocytes analysed is indicated. Images on the right are magnifications of the region indicated by the *white square*. On the right the corresponding quantification of the Sgo2 signal relative to CREST is shown. **d**–**f** same as **a**–**c** respectively for oocytes injected with control IgG or Bub1 antibody. In **d**, *arrowheads* indicate single sisters. For **b**, **e**, *n* number of kinetochore pairs analysed, mean and *error bars* ± s.d. are indicated, from two independent experiments, using Student’s *t*-test. Mean values are indicated in *red* for each condition. In histogram of **c** and **f** values indicate the mean of normalised intensities per oocyte, with *error bars* ± s.e.m. from two independent experiments, using Student’s *t*-test. *n.s*. not significant; ***P < 0.0001. A.U., arbitrary units. *Scale bars*: 5 μm

It was assumed that Bub1 kinase activity is required for Sgo2 localisation, similar to it being required for mitotic Sgo1 localisation. Conditional knock-out of *Bub1* leads to gross chromosome missegregations in oocytes because of accelerated meiosis I progression and SAC inactivation, and additionally, to the presence of single sister chromatids^27^, indicating that Bub1 protein is required for some aspects of centromeric cohesin protection in meiosis. To get insights into the requirements of the kinase activity of Bub1 for cohesin protection we analysed oocytes from *Bub1KD* mice^28^. We found that *Bub1KD* oocytes enter meiosis I normally. But most *Bub1KD* oocytes were defective in metaphase-to-anaphase transition and remained either arrested in metaphase I or underwent PB extrusion with a significant delay (Supplementary Fig. 4a). The observed arrest/delay in metaphase I was due to SAC activation, because Mps1 was enriched at kinetochores compared to controls (Supplementary Fig. 4b), and the arrest/delay was suppressed by adding Reversine (Supplementary Fig. 4c). Bub1 kinase is required for chromosome congression in somatic cells^21, 28^, and failures in chromosome congression are expected to activate the SAC, which is what we observed in *Bub1KD* oocytes. Our results indicate that similar to mitosis^37^, the kinase activity of Bub1 is not absolutely required for a functional SAC in meiosis. Because of this SAC arrest we could not analyse chromosome segregation by in vitro oocyte culture to address whether single sisters were present, as we could not obtain enough oocytes undergoing the first division. But after hormonal stimulation of adult *Bub1KD* mice and dissection of oviducts we were able to obtain enough oocytes that had overcome the SAC arrest and matured in vivo into metaphase of meiosis II. These *Bub1KD* oocytes did not show any gross chromosome missegregations such as observed upon loss of SAC control in Bub1 knock-out oocytes or other conditional SAC mutants, indicating that indeed, the meiotic SAC is functional without Bub1 kinase activity. Importantly, no increase in single sister chromatids in metaphase II, and no increase in interKT distances in metaphase II was observed in *Bub1KD* oocytes compared to controls (Fig. 3a, b). These observations are in accordance with the fact that *Bub1KD* mice are fertile^28^. Thus, our results show that Bub1 kinase activity is not essential for cohesin protection in meiosis I, at least in oocytes matured in vivo.

### Consequences of inactivation of both Mps1 and Bub1 kinases

In mitosis, correct Sgo1 localisation for centromeric cohesin protection from the prophase pathway depends on H2A-T120 phosphorylation by Bub1 kinase^17–20^. Sgo2 is different from Sgo1 not only because a large part of the Sgo2 protein sequence is not homologous to Sgo1, but also because it protects cohesin from Separase cleavage in meiosis, and not the prophase pathway, such as in mitosis (Supplementary Fig. 4d)^9, 38^. We asked whether Bub1 kinase is required for Sgo2 localisation at all. Indeed, Sgo2 recruitment to the centromere region was reduced in *Bub1KD* oocytes by around 50% compared to the control, therefore there is some requirement of Bub1 kinase for proper Sgo2 localisation (Fig. 3c). Still, there were significant amounts of Sgo2 remaining which are apparently sufficient to bring about centromeric cohesin protection. This was surprising given the fact that localisation of Sgo2 was thought to depend on Bub1 kinase activity. We asked whether inhibition of Bub1 with blocking antibody^39^ would lead to loss of Sgo2 and the separation of sister chromatids. (We could not perform morpholino-knock-down experiments due to the high stability of the protein in GV oocytes.) Such as already observed in Bub1 knock-out oocytes, injection of blocking antibody lead to an acceleration of anaphase I onset because of inactivation of the SAC (Supplementary Fig. 4e), and the presence of sister chromatids in metaphase II (Fig. 3d)^39^. Importantly, an increase in interKT distances and a reduction of Sgo2 at the centromere region was observed (Fig. 3e, f). Because no sister separation or increase of interKT distances was observed in *Bub1KD* oocytes, the result with Bub1-blocking antibodies demonstrates that Bub1 has a role for protection of centromeric cohesin independently of its kinase activity.

**Fig. 4:**
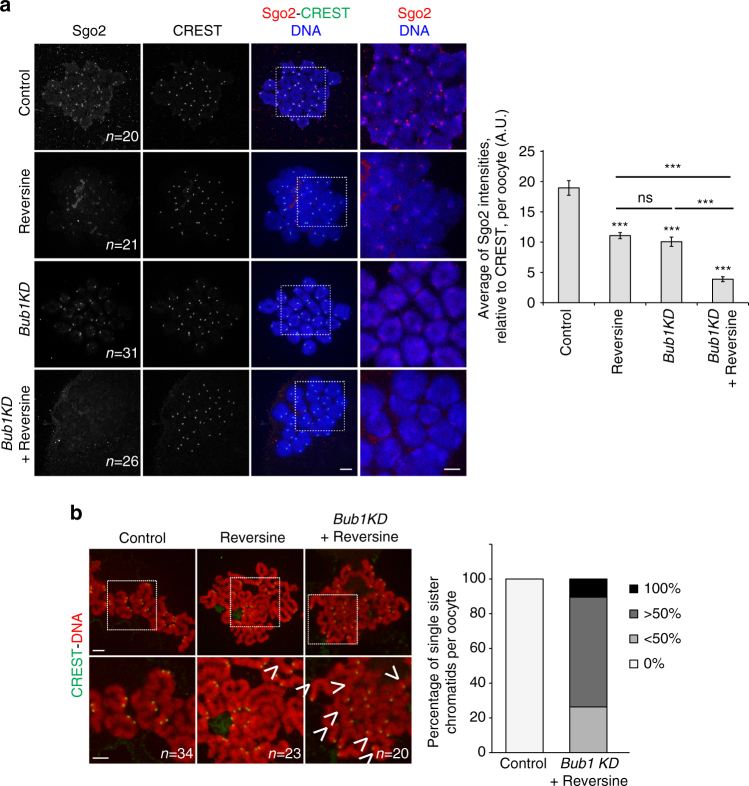
Bub1 and Mps1 kinases are both required for Sgo2 localisation and cohesin protection. **a** Chromosome spreads performed at GVBD + 3h30 on control and *Bub1KD* oocytes treated or not with Reversine (from GVBD onwards) as indicated. Chromosome spreads were stained with anti-Sgo2 antibody (*red*), and CREST anti-serum (*green*). Chromosomes were stained with Hoechst (*blue*). The number of oocytes analysed is indicated. Images on the *right* are magnifications of the region indicated by the *white square*. On the *right* the corresponding quantification of the Sgo2 signal relative to CREST is shown. Values indicate mean, *error bars* ± s.e.m. from four independent experiments, using Student’s *t*-test. (*n.s*. not significant; ***P < 0.0001). A.U., arbitrary units. **b** Metaphase II spreads of control and *Bub1KD* oocytes treated with Reversine, where indicated. Kinetochores were stained with CREST (*green*), chromosomes with Propidium iodide (*red*). *n* indicates the number of oocytes analysed, and *arrowheads* indicate single sisters. Histogram in the *right* shows the percentage of metaphase II oocytes containing 0%, <50%, more than 50, or 100% of single sister chromatids observed in five independent experiments, *scale bars*: 5 μm

To address whether Bub1 kinase activity plays a role in Mps1 kinase-dependent Sgo2 localisation, we asked whether inhibition of both Mps1 and Bub1 kinases was cumulative for loss of Sgo2. In *Bub1KD* oocytes treated with Reversine, 80% of Sgo2 is lost in the centromere region of metaphase I chromosomes compared to 40–50% in Bub1KD or Reversine-treated oocytes (Fig. 4a **)**. Activities of both kinases are therefore required for Sgo2 recruitment in oocyte meiosis I and act synergistically.

Loss of Sgo2 from the centromere region to levels that are barely detectable was expected to have severe consequences on protection of centromeric cohesin and chromosome segregation. We asked whether the phenotype in metaphase II upon loss of both Bub1 and Mps1 kinase activity was comparable to the phenotype observed upon complete loss of Sgo2. As described above, treating *Bub1KD* oocytes with Reversine rescued PB extrusion because the SAC arrest was suppressed (Supplementary Fig. 4c). This allowed us to address whether the phenotype was similar to Sgo2 knock-out oocytes, which enter meiosis II with all sister chromatids precociously separated. Simultaneous inactivation of both Mps1 and Bub1 kinases indeed resulted in 100% of metaphase II oocytes with single sister chromatids, and 75% of metaphase II oocytes with a very high number ( > 50%) of single sister chromatids or even all chromatids separated (10% of oocytes) (Fig. 4b). The phenotype is therefore quite strong, but still not as strong as upon complete loss of Sgo2, where all sisters are separated in 100% of metaphase II oocytes. This may be due to the fact that Reversine inhibition of Mps1 is not as efficient as a genetic approach, or the existence of alternative pathways for Sgo2 recruitment, such as Histone H3 phosphorylation by Haspin kinase, which plays a role for Sgo1 recruitment in mitosis, or Bub1 protein which may function as a scaffold to recruit Sgo2 independently of its kinase activity. Nevertheless, our data demonstrate that kinase activities of Mps1 and Bub1 play a predominant role for Sgo2 recruitment and cohesin protection in meiosis I.

Mps1 and Aurora B kinase activities are interdependent in mitosis. In oocytes, Aurora B and C are required for correction of erroneous microtubule attachments and correct chromosome segregation^40–42^, and Aurora B/C localisation depends on Mps1 recruitment to kinetochores^32^. We asked whether reversely, kinetochore localisation of Mps1 depends on Aurora B/C kinase activity. Oocytes treated with the well-characterised Aurora B/C inhibitor ZM447439 had reduced Mps1 levels at kinetochores, but Sgo2 localisation was only slightly diminished (Supplementary Fig. 5a, b). Inhibition of Aurora B/C led to defects in cytokinesis, prohibiting further analysis of chromosome segregation. We conclude that efficient Sgo2 recruitment requires mainly Mps1 activity, but Aurora B/C is required for full Mps1 activity and therefore participates in Sgo2 recruitment.

### Two pools of Sgo2 that are regulated differently

**Fig. 5:**
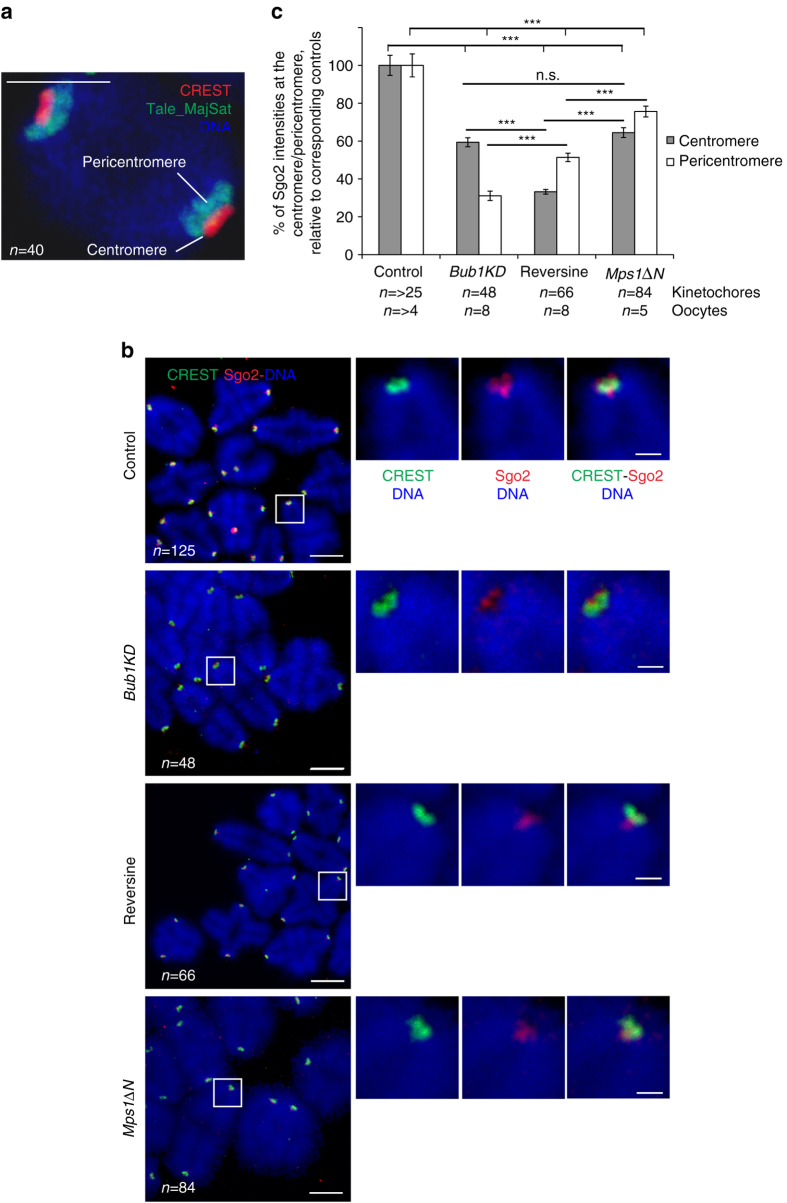
Bub1 and Mps1 kinases localise Sgo2 preferentially within the pericentromere and to the centromere, respectively. **a** Chromosomes spreads were performed from oocytes injected with mRNA coding Tale-MajSat GFP (marker of the pericentromere) and stained with anti-GFP antibody (*green* = pericentromere) and CREST anti-serum (*red* = centromere). Chromosomes were stained with Hoechst (*blue*). The different regions stained are indicated, refer also to the corresponding spreads in Supplementary Fig. 6a. *Scale bar*: 2.5 μm **b** Chromosome spreads were performed at GVBD + 6h of control, *Bub1KD*, Reversine-treated and *Mps1∆N* oocytes. MG132 was added to Reversine-treated oocytes and *Mps1∆N* oocytes at BD + 3h to prevent early anaphase onset and maintain them without entering anaphase I until BD + 6h. Chromosome spreads were stained with anti-Sgo2 antibody (*red*), and CREST anti-serum (*green*). Chromosomes were stained with Hoechst (*blue*). Images on the *right* are magnifications of the region indicated by the *white square*. *n* number of kinetochore pairs analysed. *Scale bars*: 5 μm in whole spreads and 1 µm in magnified images. **c** Mean of Sgo2/CREST intensities at the centromere vs. pericentromere in *Bub1KD*, Reversine-treated and *Mps1∆N* oocytes were normalised on the mean Sgo2/CREST in control oocytes (with or without MG132). Number of kinetochore pairs and number of oocytes analysed are indicated. Values indicate mean, *error bars* ± s.e.m. from one representative experiment performed at least in duplicate, using Student’s *t*-test. (*n.s*. not significant; ***P < 0.0001)

How can we reconcile the fact that both Mps1 and Bub1 kinase activities are required for Sgo2 localisation, but only inhibition of Mps1 led to increased interKT distances in meiosis II and precocious sister separation? We hypothesised that Mps1 and Bub1 kinases recruit Sgo2 preferentially to different centromere regions, which are not equally important for cohesin protection. To address this point we first determined where centromere and pericentromere regions are exactly localised on metaphase I chromosome spreads. We injected mRNAs coding for a fluorescent TALE recognising the major satellite repeats of the pericentromere (Tale_MajSat)^43^ in GV oocytes, allowed oocytes to progress into metaphase I, where they were fixed and stained with anti-GFP antibody and anti-CREST serum, which recognises the centromere. Figure 5a shows a bivalent of a representative chromosome spread (Supplementary Fig. 6a) to illustrate where the pericentromere is found relative to the centromere, on metaphase I mono-oriented sister kinetochore pairs. Importantly, pericentromere and centromere can be well distinguished with our image acquisition protocol. To address whether distinct pools of Sgo2 can be detected, we analysed chromosome spreads of control, Reversine-treated, and *Bub1KD* metaphase I oocytes at GVBD + 6h, with high-resolution confocal microscopy. Indeed, we found that in the control, Sgo2 staining was distinctly overlapping with the centromere and also localised between the sisters within the pericentromere region (Fig. 5b, Supplementary Fig. 6b). In Reversine-treated, and *Bub1KD* oocytes, we observed a reduction of both populations of Sgo2. Importantly, we found that loss of Bub1 kinase activity decreased foremost Sgo2 between sisters within the pericentromere, whereas loss of Mps1 kinase decreased Sgo2 more at the centromere than the pericentromere (Fig. 5b, c). 3D rendering using Arivis Vision 4D software of individual bivalents is shown to better visualise the different Sgo2 pools (Supplementary Fig. 6b). Inactivation of Mps1 kinase affected therefore mostly the Sgo2 pool at the centromere, leading to loss of cohesin protection. Our data indicate that the centromeric pool of Sgo2 is required for cohesin protection, because loss of pericentromeric Sgo2 between sisters did not lead to precocious sister chromatid separation in *Bub1KD* oocytes.

Our results predicted that in *Mps1ΔN* oocytes, which do not precociously separate sister chromatids, Sgo2 at the centromere would still be present. Indeed, even though Sgo2 levels are decreased in *Mps1ΔN* oocytes, amounts of Sgo2 at the centromere are not significantly different from those in Bub1KD oocytes at the centromere (Fig. 5b, c, Supplementary Fig. 6b). We conclude that Mps1 kinase activity is required for Sgo2 localisation at the centromere, which brings about centromeric cohesin protection.

### Bub1-dependent H2AT121 phosphorylation is dispensable for protection

**Fig. 6:**
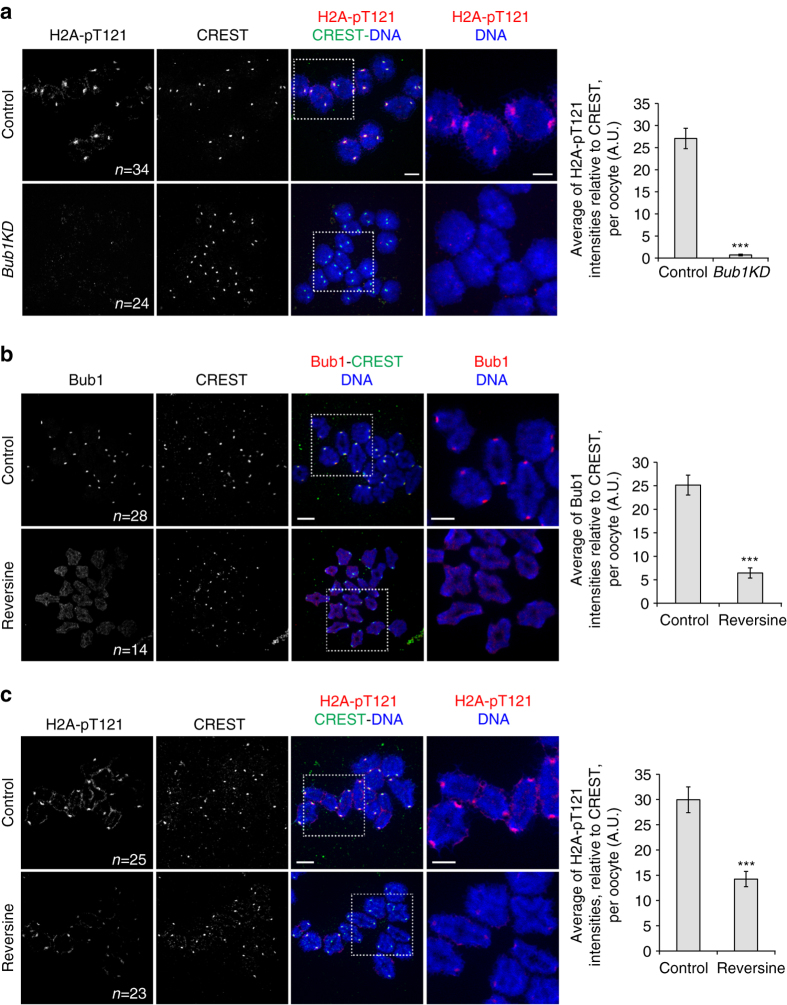
Mps1 kinase activity contributes to Bub1-dependent H2A T121 phosphorylation. Chromosome spreads were performed at GVBD + 3h30 from control and *Bub1KD* oocytes in **a** and control and Reversine-treated oocytes in **b**, **c** and stained with anti-H2A-pT121 antibody (*red*) in **a**, **c** or with anti-Bub1 antibody (*red*) in **b**. For **a**–**c**, centromeres were stained with CREST anti-serum (*green*) and chromosomes with Hoechst (*blue*). Images in the right are magnification of the region indicated by the *white square*. *n* number of oocytes analysed. On the *right* the corresponding quantifications of the H2A-pT121 signal relative to CREST in **a** and **c** or Bub1 signal relative to CREST in **b** are shown. In each histogram of this figure, values indicate mean, *error bars* ± s.e.m. from two **a** or five **b** and **c** independent experiments, using Student’s *t*-test. (***P < 0.0001). *A.U*. arbitrary units. *Scale bars*: 5 μm

We were wondering whether Bub1 kinase is required at all for H2A-T121 phosphorylation (corresponding to T120 in human H2A) in oocyte meiosis I. Our data show that this Histone mark is undetectable in *Bub1KD* oocytes (Fig. 6a), demonstrating that this mark is exclusively generated in a Bub1 kinase-dependent manner. As significant amounts of Sgo2 remained at the centromere in *Bub1KD* oocytes, we conclude that Sgo2 localisation for cohesin protection occurs independently of H2A-T121 phosphorylation, which is also confirmed by the fact that *Bub1KD* mice are fertile.

**Fig. 7:**
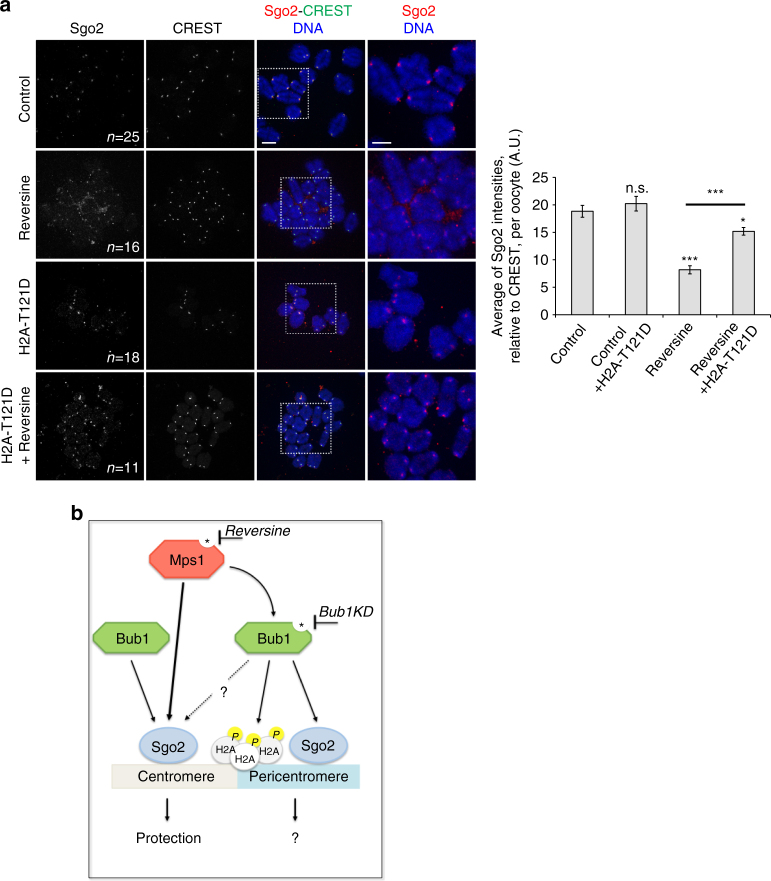
A phospho-mimicking H2A-T121D partially rescues Sgo2 localisation in Reversine oocytes. **a** Control and Reversine-treated oocytes (from GVBD onwards), were injected with mRNAs encoding H2A-T121D (at GV stage), fixed in metaphase I and stained with anti-Sgo2 antibody (*red*) and CREST anti-serum (*green*). Chromosomes were stained with Hoechst (*blue*). Images on the *right* are magnifications of the region indicated by the *white square*. *n* number of oocytes analysed. Corresponding quantification is shown on the *right*. Values indicate mean, *error bars* ± s.e.m. from three independent experiments, using Student’s *t*-test. (*n.s*. not significant; *P < 0.05, ***P < 0.0001). A.U., arbitrary units. *Scale bars*: 5 μm. **b** Model of how Sgo2 recruitment to centromeres and within the pericentromere may occur in oocytes. Bub1 kinase localises Sgo2 at the pericentromere by generating the Histone H2ApT121 mark, with the participation of Mps1 kinase, which is required for Bub1 kinetochore recruitment. Mps1 kinase additionally localises Sgo2 to the centromere for Cohesin protection, independently of H2A-T121 phosphorylation. Bub1 protein participates in Sgo2 recruitment to the centromere. Kinetochore localisation of Mps1 is not required for Sgo2 recruitment to the centromere and cohesin protection. Sgo2 at the pericentromere may be required for other functions of Sgo2 not related to cohesion protection. See text for details

As both centromeric and pericentromeric Sgo2 are reduced in Mps1 kinase-deficient oocytes, we asked whether Mps1 kinase activity was required for Bub1 localisation and subsequent H2A phosphorylation to localise Sgo2 at the pericentromere. Indeed, Bub1 was strongly reduced at kinetochores in oocytes treated with Reversine (Fig. 6b). H2A-T121 phosphorylation was reduced to half in Reversine-treated oocytes (Fig. 6c), indicating that small amounts of Bub1 at kinetochores can still phosphorylate H2A, or alternatively, phosphorylation of H2A by Bub1 can also take place in the cytoplasm because of high turn-over rates of H2A at the centromere. If Mps1 localises Sgo2 through Bub1-dependent H2A phosphorylation, expression of a phosphomimicking mutant of H2A (H2A-T121D) should rescue Sgo2 localisation in Reversine-treated oocytes, and this was indeed the case (Fig. 7a). We conclude that Mps1 kinase is essential for the recruitment of Sgo2 to the centromere independently of this Histone mark for cohesin protection, but additionally participates in H2ApT121-dependent recruitment of Sgo2, potentially for other functions such as SAC control or regulation of pulling forces of the spindle and chromosome congression^9^.

## Discussion

Mammalian female meiosis is highly error prone, which has severe consequences for producing healthy offspring. Failure to maintain sister chromatids together correctly throughout the first meiotic division leads to their segregation at random in anaphase II, as single sisters cannot correctly attach to the spindle and properly align at the metaphase plate in meiosis II. Precocious loss of cohesin with age is thought to contribute to the high error rate in oocyte chromosome segregation^34^. Accordingly, knock-out mice harbouring oocytes that are impaired in maintaining centromeric cohesion are sterile, showing the crucial role of cohesin protection for generating healthy oocytes of correct ploidy that can be fertilised and that support further embryo development^4, 44^.

The Shugoshin protein family is thought to be localised to the centromeric region through several pathways working in parallel^45^. In mitosis, localisation of Sgo1 at the centromere depends on Bub1-dependent H2A-T120 (or murine T121) phosphorylation^17–21^. Additionally, Haspin kinase-dependent Histone H3T3 phosphorylation for recruitment of the Chromosomal Passenger Complex, and HP1 (Heterochromatin protein 1) stabilise Sgo1 binding to the centromere, for correct protection of cohesin from prophase pathway-dependent removal^45^. The role of Sgo2 at the centromere for cohesin protection in mitosis is unknown, and it has been shown that mitotic Sgo2 recruitment is regulated in an unknown manner, different from Sgo1^29, 30^. In mammalian meiosis, Sgo2 is essential for centromeric cohesin protection, and it was therefore important to determine how Sgo2 localisation is regulated^4^. Here we identified two mechanisms for Sgo2 recruitment in mammalian oocyte meiosis. Both require Mps1 kinase activity, but only one is identical with the known Bub1-dependent generation of the H2A-pT121 mark to localise Sgo1 in mitosis^17–20, 29, 30^. Reversine-treated oocytes where Mps1 kinase activity is inhibited, are defective in both mechanisms, even though a small amount of Sgo2 remaining in the centromere region is still enough to ensure some cohesin protection. InterKT distances of sister chromatid pairs in meiosis II were increased, and single sister chromatids were observed as oocytes entered meiosis II. Our results indicate that Sgo2 recruitment is regulated differently from Sgo1 in *Saccharomyces cerevisiae*, which does not required Mps1 activity for protection of centromeric cohesin in anaphase I^46^.

Our data indicated that separate pools of Sgo2 exist at centromeres in meiosis I. With optimised confocal imaging to obtain near super-resolution images we were able to distinguish at least two pools of endogenous Sgo2 in metaphase I, at the centromere and between sisters at the pericentromere, with the pool at the centromere being foremost required for cohesin protection (Fig. 7c). Our data support the conclusion that Sgo2 localisation to the centromere region requires Bub1 protein, but can occur without Bub1 kinase activity, and crucially, without H2A-T121 phosphorylation. Sgo2 localisation to the pericentromere requires Bub1 kinase activity and H2A-T121 phosphorylation. Mps1 kinase activity is important for the localisation of both pools of Sgo2. This is in agreement with the fact that *Bub1KD* mice are fertile and can generate oocytes of the correct ploidy. Mps1 and Bub1 bring about Sgo2 recruitment independently of a functional checkpoint, because loss of another essential SAC protein, namely BubR1, does not lead to loss of Sgo2 or sister separation.

If Mps1 activity were required for localising both pools of Sgo2 it remained puzzling why not all Sgo2 was lost upon inhibition of Mps1, and only additional inhibition of Bub1 led to near complete loss of Sgo2. We think that this is due to the fact that not all Bub1 was lost from kinetochores upon Mps1 inhibition. In mitosis, Bub1 was shown to be constitutively active and to bind to Knl1 at the kinetochore, that has been phosphorylated by Mps1. Without Mps1 activity, Bub1 localisation in oocytes was indeed significantly decreased at kinetochores (Fig. 6b). Because Bub1 autophosphorylates and is supposedly active, this reduced amounts of Bub1 resulted in an only 50% decrease of H2ApT121. Therefore, Mps1 kinase activity is required for full Bub1 activity at kinetochores, but Mps1 is not absolutely essential for Bub1 to function there, explaining the additive effect on Sgo2 localisation upon inhibition of both kinases.

The presence of single sister chromatids on anaphase I spindles in oocytes devoid of Mps1 kinase activity was unlike the phenotype observed in Sgo2 knock-out oocytes. Bipolar, lateral and merotelic instead of monopolar attachments at the time of accelerated anaphase I onset in Reversine-treated oocytes may have been the reason for the detection of single sisters already in anaphase I. It has been proposed that Sgo2 is removed from the inner centromeric region in meiosis II through the bipolar tension applied on the two sister kinetochores^8, 14^. Indeed, a significant number of kinetochores harboured merotelic attachments at the time when oocytes without Mps1 undergo metaphase-to-anaphase transition. We cannot exclude that Mps1 kinase is required for monopolar orientation in meiosis I, but we do not think that this alone would lead to precocious separation of sister chromatids we observed here for the following reasons: (1) most merotelic attachments are lateral, not end-on attachments. Chromosomes in prometaphase, when accelerated anaphase I takes place, are not yet stretched as much as in metaphase I, which is supposed to induce loss of Sgo2 and loss of protection such as proposed for bipolar attached chromosomes in metaphase I^47, 48^. (2) We did not observe a further loss of Sgo2 in Reversine-treated oocytes in metaphase I that were prevented from entering anaphase I and which harbour chromosomes that are much more stretched (compare Fig. 2a, b with Supplementary Fig. 3c). (3) Tension-induced Sgo2 redistribution is thought to take place as oocytes and spermatozoa enter meiosis II^8, 14^. It concerns the pool of Sgo2 that is distinct from Sgo2 at the centromere, which we show here as being important for protection at anaphase I onset. (4) From all the checkpoint components whose loss has been studied in oocytes, only complete loss of Bub1^27^ and inhibition of Mps1 kinase activity (our study) led to precocious sister chromatid segregation, even though in all cases^27, 32, 33, 49–51^ accelerated anaphase I onset at a time when attachments are not correct, occurred. (5) *Mps1ΔN* oocytes, which show exactly the same phenotype as Reversine-treated oocytes as far as SAC control is concerned^32, 33^, do not separate sister chromatids precociously. Hence it is attractive to speculate that Mps1 at the kinetochore may directly phosphorylate and thereby regulate Sgo2 for its function in cohesin protection in oocyte meiosis. Accelerated anaphase I onset in Reversine-treated oocytes at a time when attachment errors are present together with loss of cohesin protection also explain why we observed single sister chromatids on anaphase I spindles.

Our work shows that protection of cohesin from prophase pathway-dependent removal by Sgo1 in mitosis, and protection from Separase-dependent removal by Sgo2 in meiosis are regulated in a distinct manner. In addition to its role in the SAC, Mps1 has a meiosis-specific role in Sgo2 recruitment, which is essential for correct chromosome segregation. Future work will show how Mps1 brings about Sgo2 recruitment preferentially to the centromere in meiosis I. Our results open up new lines of investigation, it will for example be interesting to determine whether Sgo2 recruited by Bub1 kinase is foremost required for other key functions of Sgo2, such as tension sensing, SAC inactivation, and control of pulling forces^9^. Our work here provides new insights into the molecular pathways required for correct chromosome segregation in mammalian oocytes, which will help us better understand how errors can arise also in human oocytes.

## Methods

### Mouse oocytes harvesting, culture, and inhibitors

All animal experiments were subjected to ethical review and done under the authorisation B-75-1308, according to current French guidelines. Adult CD-1 and C57BL/6 mice (*control, Bub1KD, BubR1−/−*, *Mps1ΔN)* were sacrificed, and ovaries dissected to obtain oocytes at the GV (Germinal Vesicle stage). Only oocytes undergoing GVBD up to 90 min after release were used. Pools of oocytes undergoing GVBD at the same time ( ± 15 min) were used for experiments. The Mps1 inhibitor Reversine (Cayman Chemical Research, 10004412) was added at GVBD at a final concentration of 0.5 μM. MG132 (Sigma-Aldrich, C2211) was added at GVBD + 3h at a final concentration of 20 μM and ZM447439 (Tocris, 2458) at GVBD at a final concentration of 10 μM. Oocytes were cultured in self-made M2 medium without CO_2_. In vivo matured oocytes were obtained by injecting mice intraperitoneally with 5 UI of pregnant mare’s serum (PMS, Intervet) and 48 h later with 5 UI of human gonadotrophin (Intervet). Metaphase II oocytes surrounded by follicular cells were collected from the oviduct and incubated in M2 medium. Oocytes were separated from the follicular cells by adding hyaluronidase (Sigma-Aldrich, H4272). The zona pellucida was removed by exposing oocytes to low pH of tyrode acid solution^29^, for both chromosome spread and whole-mount oocyte staining.

### Plasmids

Reverse transcription was done on total mouse fibroblast RNA using the SuperscriptIII CellsDirect Kit (Invitrogen, 46-6320). The following primers were used to amplify the full-length coding sequence of mouse Histone H2A by PCR: 5ʹ-ATATCTCGAGCCATGTCCGGTCGT-3ʹ, 5ʹ-CGGGGATCCTCACTTGCCCTTCG-3ʹ. Histone H2A was PCR cloned into pRN3 introducing a GFP-tag at the Nʹ terminus. This construct was used to generate Histone H2A-T121D, using the following primers: 5ʹ-TGCTGCCCAAGAAGGACGAGAGCCACCAT-3ʹ, 5ʹ-ATGGTGGCTCTCGTCCTTCTTGGGCAGCA-3ʹ. TALE-mClover against mouse major satellite sequence expressing plasmid was obtained from Addgene (pTALYM3B15, deposited by Maria-Elena Torres-Padilla).

### In vitro translation and microinjection

In vitro translation of mRNAs was performed with the Ambion mMessage Machine Kit. mRNA purified on RNAeasy columns (Qiagen) was microinjected with Eppendorf micromanipulators on a Nikon Eclipse Ti microscope and a FemtoJet microinjector^29, 30^. Oocytes were kept arrested at GV stage in M2 medium with 100 μg/ml of dibutyl cyclic AMP (dbcAMP) for 3 h after injection for protein expression. Bub1 blocking antibody was injected at GV, oocytes were released 2 h after injection, and invalidation of Bub1 was controlled by assessing time of PB extrusion. For Mps1 knock-down experiments, translation-blocking antisense Morpholino oligonucleotides 5ʹ CAATTAACTCTTCAGCCTCCATTTC 3ʹ (Gene Tools) were injected at a concentration of 300 nM with constant flow to deplete Mps1. After injection, oocytes were kept arrested at GV stage in M2 medium supplemented with 100 μg/ml dbcAMP for 24 h, during which time the medium was renewed twice.

### Immunofluorescence

To harvest prometaphase I and metaphase I oocytes, controls and Bub1 deficient oocytes were fixed at 4 or 6 h after GVBD, respectively. Reversine-treated oocytes (which are strongly accelerated in meiotic maturation^33^) were fixed at 3.5 h after GVBD, which corresponds to 30–60 min before PB extrusion, or treated with MG132 at GVBD + 3h, and fixed at GVBD + 6h. Metaphase II oocytes were fixed 14–16 h after GVBD for all conditions. For chromosome spreads, oocytes were fixed in 1% paraformaldehyde (Sigma-Aldrich, 441244), 0.15% Triton-X (Sigma-Aldrich, T8787), and 3 mM DTT (Sigma-Aldrich, D9779). Oocytes for whole-mount staining of spindle microtubules were incubated in a cold treatment solution (0.08 M PIPES, 1 mM MgCl_2_, pH7.4) on top of an ice-water bath to maintain only stable microtubule fibres, and directly fixed in BRB 80^29^ containing 0.3% Triton-100 and 1.9% formaldehyde (Sigma-Aldrich, F1635). After several washes, oocytes were incubated in PBS containing 3% BSA and 0.1% Triton X-100 overnight at 4 °C. Antibodies were used at the following concentrations: polyclonal rabbit Mps1 antibody (gift from Hongtao Yu, 1/100), mouse monoclonal Bub1 antibody (gift from S. Taylor, 1/50), polyclonal rabbit Sgo2 antibody (gift from José Luis Barbero, 1/50), mouse monoclonal α-tubulin (DMA1) coupled to FITC (Sigma-Aldrich, F2168; 1/100), human CREST auto-immune antibody (Cellon SA, HCT-0100; at 1/100), and polyclonal rabbit antibody H2A-pT121 (Active Motif, #39391; at 1/2500 with 5% BSA). The following secondary antibodies were used: Cy3 anti-rabbit (Jackson ImmunoResearch, 711.166.152; 1/200), Cy3 anti-mouse (Jackson ImmunoResearch, 715.166.151; 1/200), and anti-human Alexa 488 (Life Technologies, A11013; 1/200). To stain chromosomes, Hoechst 33342 (Invitrogen, H21492) or propidium iodide (Sigma, P4864) was added for 10 min at a concentration of 0.1 mg/ml and 0.05 mg/ml, respectively, before mounting chambers in AF1 (Citifluor, UK). For detection of pericentromeric chromatin, GV oocytes were injected with mRNA expressing Tale_MajSat, allowing detection of the pericentromere with anti-GFP antibody (Cell Signaling, 1/50).

### Image acquisition and treatment

Acquisitions of stained chromosome spreads for quantifications were done with a Zeiss spinning disc confocal microscope, unless otherwise stated. *Z*-stacks (0.4 μm intervals) were acquired using a ×100/1.4 Oil Dic objective and Metamorph software. Image processing and quantification were performed with ImageJ software. Fluorescence intensity of signals at centromeres was calculated using 10 × 10 (12 × 12 for H2ApT121 staining) pixel boxes that were manually placed over centromeres. Summed pixel values of a given channel were calculated. Another 10 × 10 (12 × 12 for H2ApT121) staining box was placed adjacent to this to calculate background signal, which was then subtracted from the centromere signal. All stainings were normalised to the CREST signal on the same kinetochore. For spindle stainings (Fig. 1f) and high-resolution chromosome spreads (Fig. 5) acquisitions were done on a Leica TCS SP5-II, using a Leica ×63 oil immersion objective (HCX Plan APO CS, NA 1.4), with an optimised workflow with refractive index matching mounting medium^52^, except that acquisitions of chromosome spreads were not deconvoluted. In total, 150–200 *z*-sections of 0.08 μm were taken for acquisitions of spindle stainings in whole mount oocytes and 30–40 sections of 0.08 μm for chromosome spreads. The *z*-stacks were used for 3D rendering using Arivis Vision4D, without prior deconvolution; spindle stainings were deconvoluted with Huygens 3.7 software^52^. Fluorescence intensity of signals at centromeres and pericentromere was calculated using 12 pixels diameter circles that were manually placed over centromeres and pericentromere. The summed pixel values of a given channel were calculated. For each circle, another circle was placed adjacent to calculate background signal, which was then subtracted from the centromere/pericentromere signal. All stainings were normalised to the CREST signal on the same kinetochore.

For live imaging, a motorised inverted Nikon TE2000E microscope with a Plan APO ×20/0.75 NA objective and equipped with a PrecisExite High Power LED Fluorescence (LAM 1: 400/465, LAM 2: 585), a heating block (PECON Temp controller 2000-2), a Märzhäuser Scanning Stage, a CCD camera (Photometrics), and controlled by Metamorph software was used.

### Statistical analysis

All experiments were performed at least in duplicate. Shown are results from all experiments performed (except for Fig. 5 where only one representative experiment have been used for graph quantification). For quantifications of intensities per oocyte, 10–20 kinetochore pairs were quantified per oocyte. In each histogram, values correspond to the mean ± s.e.m. calculated on a minimum of eight oocytes. Scatter dot plots were obtained with PRISM6 software and represent all kinetochore pairs quantified. Mean ± s.d. is indicated in each scatter dot plot. In all experiments, statistical analysis was performed by Student’s *t*-test, (n.s.: not significant; *P < 0.05, **P < 0.001, ***P < 0.0001).

### Data availability

All relevant data are available from the authors.

## Electronic supplementary material

Supplementary Information
