# Supplementary material for: Mps1 kinase-dependent Sgo2 centromere localisation mediates cohesin protection in mouse oocyte meiosis I
Source: Nat Commun. 2017 Sep 25;8:694. doi: 10.1038/s41467-017-00774-3 (PMC5612927; doi:10.1038/s41467-017-00774-3)
Supplement: Supplementary file 1 — Supplementary Information [file 41467_2017_774_MOESM1_ESM.pdf]

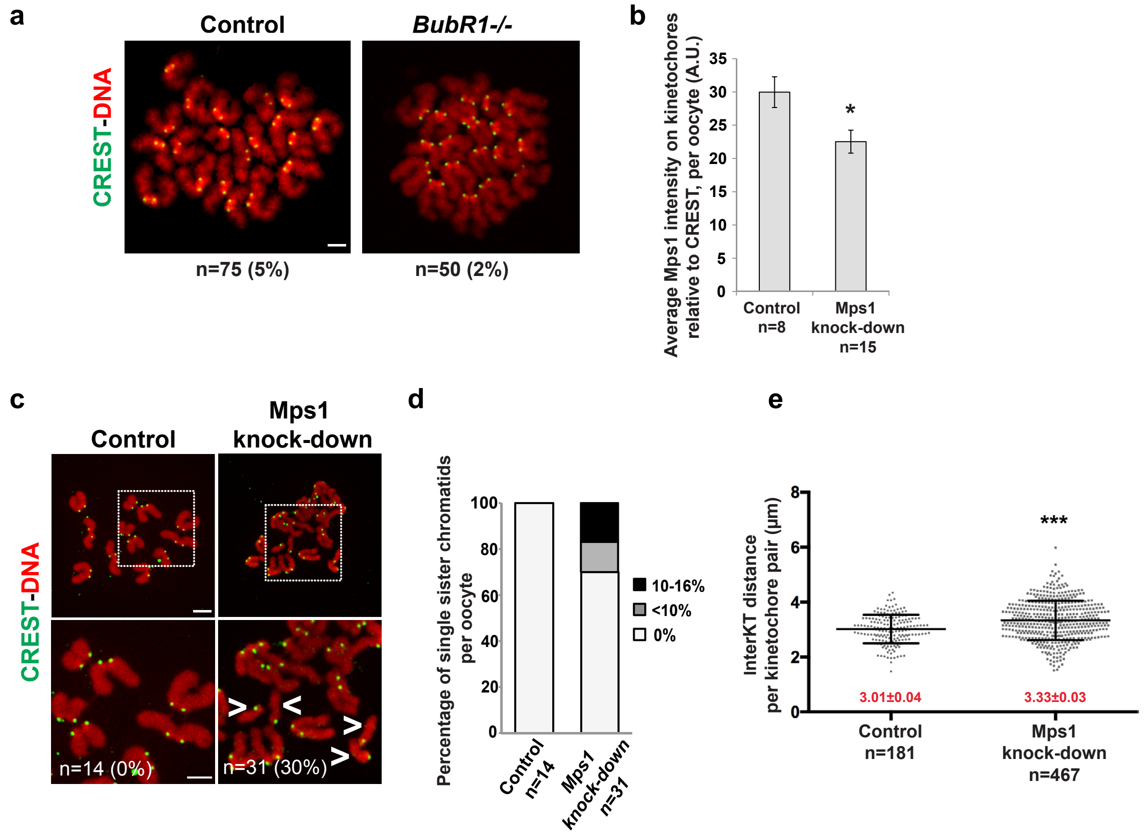

Supplementary Figure 1

Mps1, but not BubR1, is required for cohesin protection. a) Control and *BubR1* oocytes were fixed for chromosome spreads after PB extrusion in meiosis II. **b)** Morpholino-knock down efficiencies for Mps1 were controlled by immunofluorescence staining of endogenous Mps1. Histogram shows the mean per oocyte of Mps1 intensities normalized on CREST, error bars ± s.e.m. from 2 independent experiments using Student's *t*-test**.** (**P*<0.05). n: number of oocytes. A.U.: Arbitrary units. **c)** Control oocytes and oocytes injected with Mps1 morpholinos were fixed for chromosome spreads after PB extrusion in meiosis II. Chromosomes were stained with Propidium iodide (red), kinetochores with CREST (green). Images below are magnifications of the region indicated by the white square. Arrowheads indicate single sisters. n: number of oocytes analysed. Percentage of oocytes with single sisters is indicated. d) Percentage of metaphase II oocytes containing 0%, less than 10%, between 10% and 16% of single sister chromatids observed in images from c). e) Scatter dot plot showing quantification of interKT distances in metaphase II control oocytes and oocytes injected with Mps1 morpholinos is shown. Only kinetochores of intact dyads (not already separated) were used for measurements. Mean and error bars ± S.D using Student's *t*-test (****P*<0.0001) are indicated. Mean values are indicated in red for each condition n: number of kinetochore pairs analysed. 3 independent experiments were performed for c) d) and e). Scale bars: 5 µm.

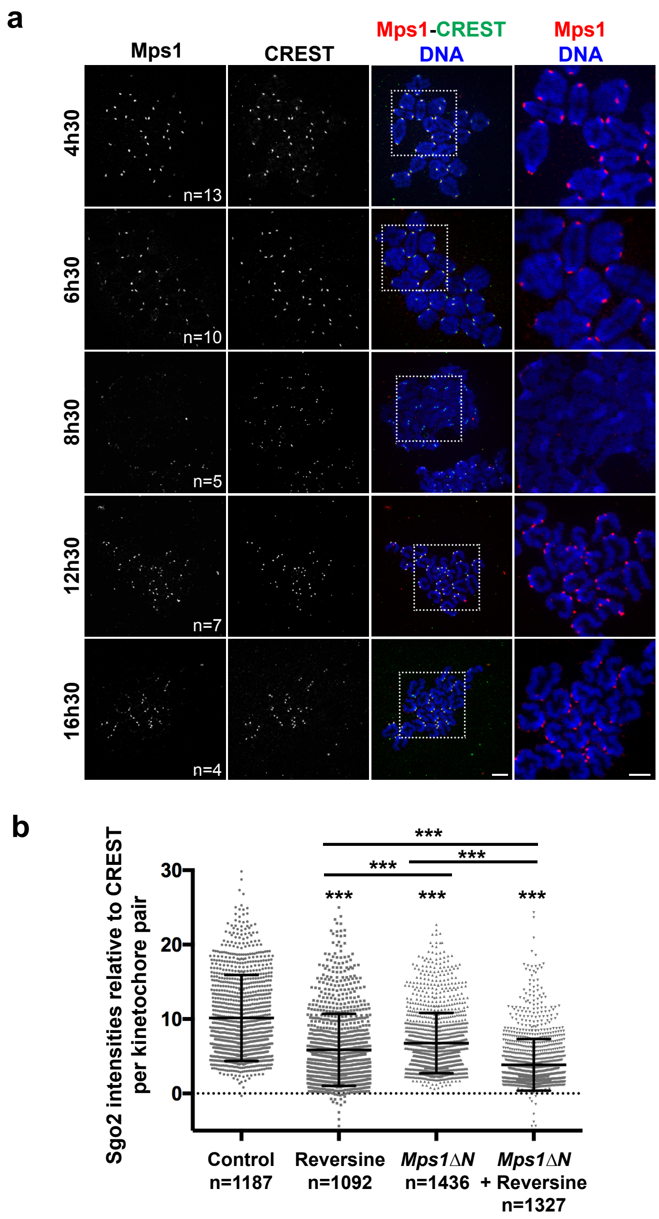

Supplementary Figure 2

Kinetochore localisation of endogenous Mps1 during meiotic maturation and Sgo2 recruitment by Mps1 kinase activity. a) Endogenous Mps1 was detected in chromosome spreads with anti--Mps1 antibody staining (red), kinetochores with CREST (green), chromosomes with Hoechst (blue). Time points after entry into meiosis (GVBD) and number of oocytes analysed are indicated. Mps1 is localised to kinetochores in prometaphase I (4h30), metaphase I (6h30), early and late metaphase II (12h30 and 16h30, respectively), but not in anaphase I (8h30). Scale bars: 5 µm. **b)** Scatter dot plot corresponding to quantifications per oocyte in **Fig. 2a**, showing quantification of the Sgo2 signal relative to CREST staining for each kinetochore pair of control, Reversine, *Mps1ΔN* and *Mps1ΔN* oocytes treated with Reversine. Mean and error bars ± S.D are indicated, using Student's *t*-test (n.s.: not significant; **P*<0.05, ***P*<0.001, ****P*<0.0001). A.U.: Arbitrary units.

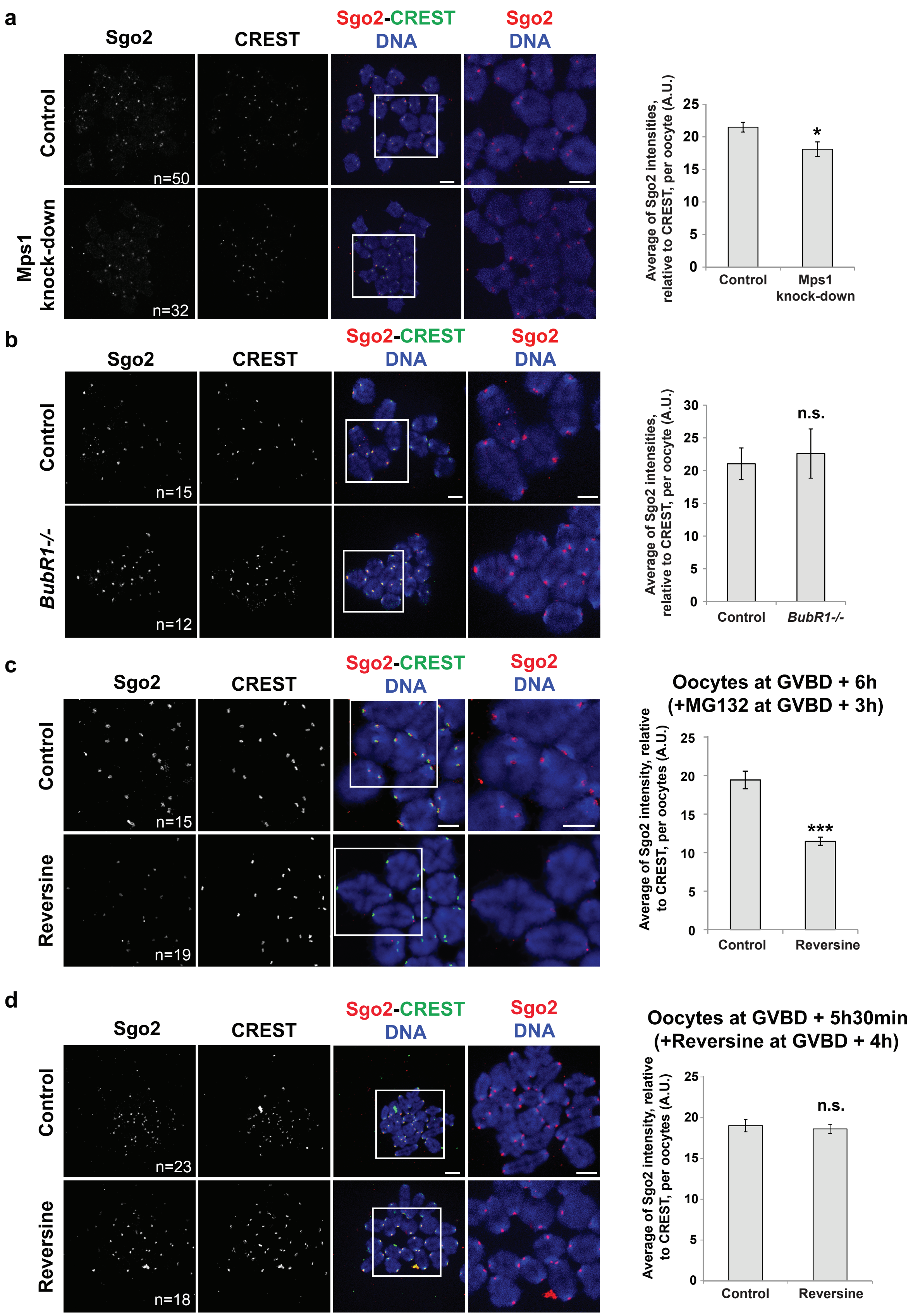

a
Sgo2
CREST
Sgo2-CREST
DNA
Sgo2
DNA
Control
Mps1 knock-down
n=50
n=32
Average of Sgo2 intensities, relative to CREST, per oocyte (A.U.)
*
Control
Mps1 knock-down
b
Sgo2
CREST
Sgo2-CREST
DNA
Sgo2
DNA
Control
BubR1-/-
n=15
n=12
Average of Sgo2 intensities, relative to CREST, per oocyte (A.U.)
n.s.
Control
BubR1-/-
c
Sgo2
CREST
Sgo2-CREST
DNA
Sgo2
DNA
Control
Reversine
n=15
n=19
Oocytes at GVBD + 6h (+MG132 at GVBD + 3h)
Average of Sgo2 intensity, relative to CREST, per oocytes (A.U.)
***
Control
Reversine
d
Sgo2
CREST
Sgo2-CREST
DNA
Sgo2
DNA
Control
Reversine
n=23
n=18
Oocytes at GVBD + 5h30min (+Reversine at GVBD + 4h)
Average of Sgo2 intensity, relative to CREST, per oocytes (A.U.)
n.s.
Control
Reversine

Supplementary Figure 3

Mps1 and not BubR1 contributes to Sgo2 centromere recruitment. Control and **a)** oocytes injected with Mps1 morpholinos or **b)** *BubR1* oocytes were used to perform chromosome spreads at GVBD + 3h30, which were stained with antibodies against Sgo2 (red) and CREST serum (green). Chromosomes were stained with Hoechst (blue). On the right the corresponding quantification of the Sgo2 signal relative to CREST staining is shown. **C)** Control and Reversine-treated oocytes were treated with MG132 at GVBD + 3h30 and used to perform chromosome spreads at GVBD+6h, which were stained with antibodies against Sgo2 (red) and CREST serum (green). Chromosomes were stained with Hoechst (blue). **d)** Control and oocytes treated with Reversine at GVBD + 4h were used to perform chromosome spreads at GVBD + 6h, which were stained with antibodies against Sgo2 (red) and CREST serum (green). Chromosomes were stained with Hoechst (blue). On the right the corresponding quantification of the Sgo2 signal relative to CREST staining is shown. The images on the right are magnifications of the region indicated by the white square. n: number of oocytes analysed. In each histogram, values indicate mean, error bars ± s.e.m. from five (a) or three (b, c, d) independent experiments, using Student's *t*-test. (n.s.: not significant; *P<0.05, **P<0.001, ***P<0.0001). A.U.: Arbitrary units. Scale bars: 5 µm

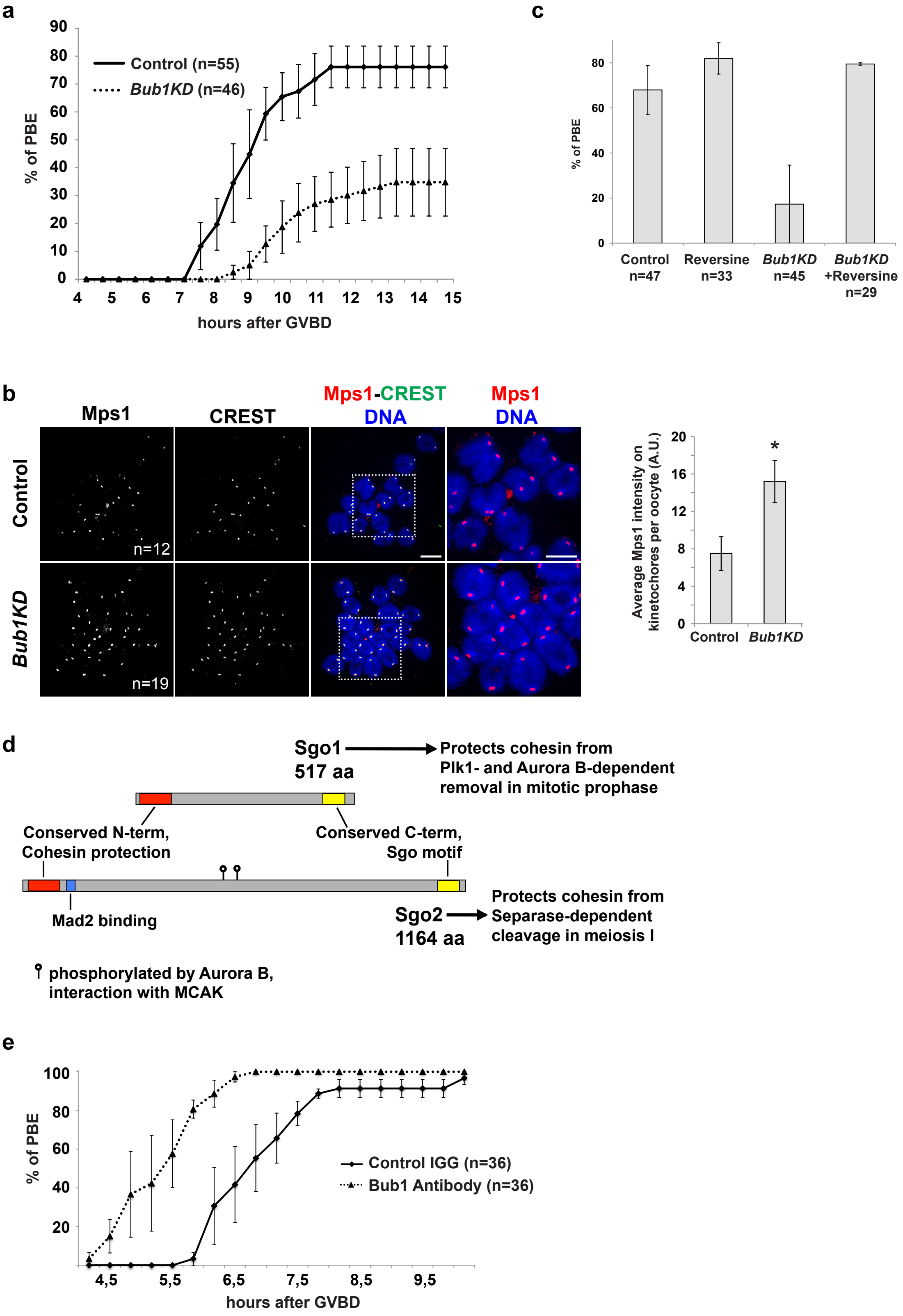
a
% of PBE
Control (n=55)
Bub1KD (n=46)
hours after GVBD
c
% of PBE
Control n=47
Reversine n=33
Bub1KD n=45
Bub1KD +Reversine n=29
b
Mps1
CREST
Mps1-CREST DNA
Mps1 DNA
Control
Bub1KD
n=12
n=19
Average Mps1 intensity on kinetochores per oocyte (A.U.)
Control
Bub1KD
*
d
Sgo1 517 aa
Protects cohesin from Plk1- and Aurora B-dependent removal in mitotic prophase
Conserved N-term, Cohesin protection
Conserved C-term, Sgo motif
Mad2 binding
Sgo2 1164 aa
Protects cohesin from Separase-dependent cleavage in meiosis I
phosphorylated by Aurora B, interaction with MCAK
e
% of PBE
Control IGG (n=36)
Bub1 Antibody (n=36)
hours after GVBD

Supplementary Figure 4

No precocious sister separation is observed in Bub1KD oocytes. a) Meiotic maturation of *in vitro* matured *Bub1KD* oocytes, compared to controls. Oocytes were released in a synchronized manner. Shown is the percentage of oocytes that were extruding the first PB at the indicated time points after GVBD. The total number of oocytes analysed from three independent experiments is indicated. **b)** Control and *Bub1KD* oocytes were used to perform chromosome spreads at GVBD + 3h30, which were stained with antibodies against Mps1 (red) and CREST serum (green). Chromosomes were stained with Hoechst (blue). On the right the corresponding quantification of the Mps1 signal relative to CREST staining is shown. In each histogram, values indicate mean, error bars ± s.e.m. from three independent experiments, using Student's *t*-test. (**P*<0.05). A.U.: Arbitrary units. Scale bars: 5 µm **C)** *Bub1KD* oocytes were treated with Reversine (from GVBD onwards), and PB extrusion was scored at 10 hours after GVBD. The indicated number of oocytes from two independent experiments was analysed. In each histogram, Values indicate mean , Error bars ± s.e.m. **d)** Comparison of mouse Sgo1 and Sgo2 protein sequences. **e)** Meiotic maturation of *in vitro* matured oocytes injected with Bub1 antibodies, compared to controls. Oocytes were released in a synchronized manner. Shown is percentage of oocytes that were extruding the first PB at the indicated time points after GVBD. The total number of oocytes analysed from three independent experiments is indicated.

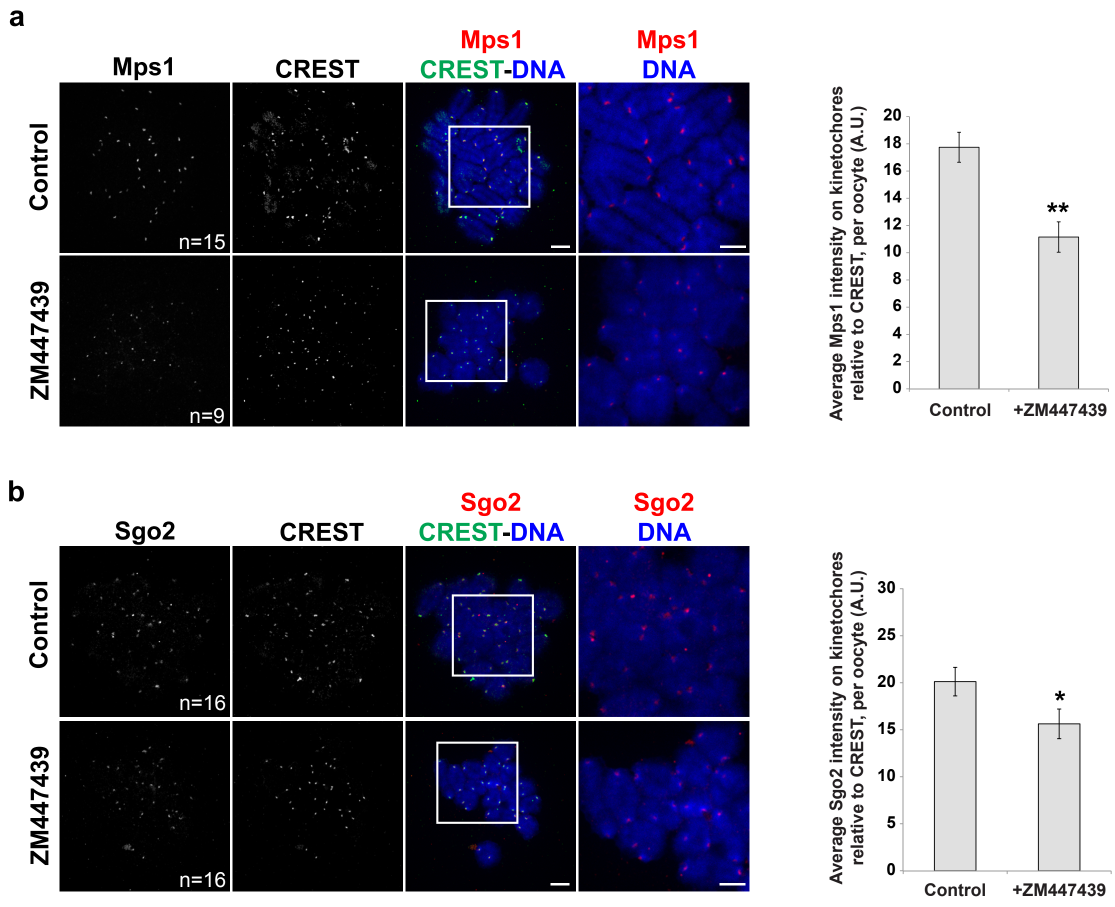

Supplementary Figure 5

Activity of Aurora B/C kinases participates in Mps1 and Sgo2 recruitment. a) Oocytes were treated with the Aurora B/C inhibitor ZM447439 where indicated. Chromosome spreads of control and ZM447439 treated oocytes were done at GVBD + 4h and stained for Mps1 (red), CREST (green), and with Hoechst (DNA, blue). White squares indicate magnifications shown on the right. Quantifications are shown in the histograms on the right. **b)** Same as in a), except that spreads were stained for Sgo2 (red). In each histogram, values indicate mean, error bars ± s.e.m. from two independent experiments, using Student's *t*-test. (n.s.: not significant; *P<0.05, **P<0.001). A.U.: Arbitrary units. Scale bars: 5 µm.

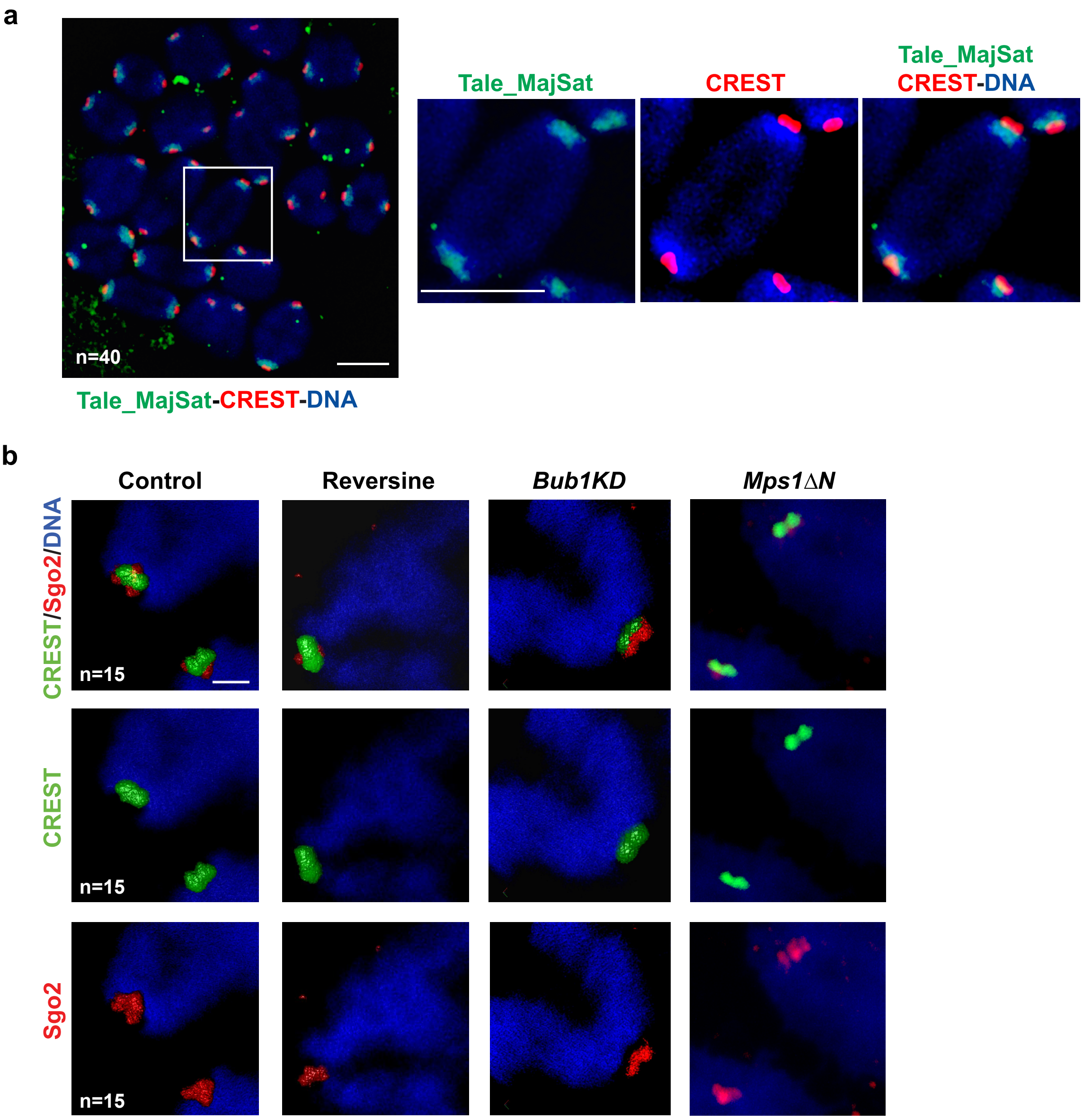

Supplementary Figure 6

Sgo2 localises to the centromere in a Mps1 kinase activity dependent manner, and to the pericentromere in a Bub1 kinase activity dependent manner. a) Chromosome spreads of Tale_MajSat GFP expressing oocytes were done at GVBD + 6h and stained with anti-CREST serum (red), GFP antibody (green), and Hoechst (DNA, blue). Number of oocytes analysed is indicated. Images on the right are magnifications of the region indicated by the white square. **b)** 3D-rendering of confocal high resolution images in Figure 5. n: number of oocytes. Scale bar: 5 µm.
